# CSF Biomarkers and Its Associations with ^18^F-AV133 Cerebral VMAT2 Binding in Parkinson’s Disease—A Preliminary Report

**DOI:** 10.1371/journal.pone.0164762

**Published:** 2016-10-20

**Authors:** Rui Gao, Guangjian Zhang, Xueqi Chen, Aimin Yang, Gwenn Smith, Dean F. Wong, Yun Zhou

**Affiliations:** 1 Department of Nuclear Medicine, the First Affiliated Hospital of Xian Jiaotong University, Xi'an, Shaanxi 710061, China; 2 The Russell H. Morgan Department of Radiology and Radiological Science, Johns Hopkins University School of Medicine, Baltimore, Maryland 21287, United States of America; 3 Department of Surgery, the First Affiliated Hospital of Xian Jiaotong University, Xi'an, Shaanxi 710061, China; 4 Department of Nuclear Medicine, Peking University First Hospital, Beijing 100034, China; 5 Division of Geriatric Psychiatry and Neuropsychiatry, Johns Hopkins Bayview Medical Center, Baltimore, Maryland 21287, United States of America; 6 Department of Psychiatry, Johns Hopkins University, Baltimore, Maryland 21205, United States of America; 7 Department of Neurology, Johns Hopkins University, Baltimore, Maryland 21205, United States of America; 8 Department of Neuroscience, Johns Hopkins University, Baltimore, Maryland 21205, United States of America; Banner Alzheimer's Institute, UNITED STATES

## Abstract

**Objective:**

Cerebrospinal fluid (CSF) biomarkers, such as α-synuclein (α-syn), amyloid beta peptide 1–42 (Aβ_1–42_), phosphorylated tau (181P) (p-tau), and total tau (t-tau), have long been associated with the development of Parkinson disease (PD) and other neurodegenerative diseases. In this investigation, we reported the assessment of CSF biomarkers and their correlations with vesicular monoamine transporter 2 (VMAT2) bindings measured with ^18^F-9-fluoropropyl-(+)-dihydrotetrabenazine (^18^F-AV133) that is being developed as a biomarker for PD. We test the hypothesis that monoaminergic degeneration was correlated with CSF biomarker levels in untreated PD patients.

**Methods:**

The available online data from the Parkinson’s Progression Markers Initiative study (PPMI) project were collected and analyzed, which include demographic information, clinical evaluations, CSF biomarkers (α-syn, Aβ_1–42_, p-tau, and t-tau), ^18^F-AV133 brain PET, and T1 weighted MRIs. Region of interest (ROI) and voxel-wise Pearson correlation between standardized uptake value ratio (SUVR) and CSF biomarkers were calculated.

**Results:**

Our major findings are: 1) Compared with controls, CSF α-syn and tau levels decreased significantly in PD; 2) α-syn was closely correlated with Aβ_1–42_ and tau in PD, especially in early-onset patients; and 3) hypothesis-driven ROI analysis found a significant negative correlation between CSF Aβ_1–42_ levels and VMAT2 densities in post cingulate, left caudate, left anterior putamen, and left ventral striatum in PDs. CSF t-tau and p-tau levels were significantly negatively related to VMAT2 SUVRs in substantia nigra and left ventral striatum, respectively. Voxel-wise analysis showed that left caudate, parahippocampal gyrus, insula and temporal lobe were negatively correlated with Aβ_1–42_. In addition, superior frontal gyrus and transverse temporal gyrus were negatively correlated with CSF p-tau levels.

**Conclusion:**

These results suggest that monoaminergic degeneration in PD is correlated with CSF biomarkers associated with cognitive impairment in neurodegenerative diseases including Alzheimer’s disease. The association between loss of dopamine synaptic function and pathologic protein accumulations in PD indicates an important role of CSF biomarkers in PD development.

## Introduction

Cerebrospinal fluid (CSF) analyses, reflecting metabolic and pathological states of the central nervous system, are widely used for Parkinson disease (PD) biomarker discovery [1–3]. Although most studies in large cohorts report a panel of CSF markers, including α-synuclein (α-syn), amyloid beta peptide 1–42 (Aβ_1–42_) and phosphorylated tau (181P) (p-tau), total tau (t-tau), were useful in distinguishing PD from controls [1, 3], the data between studies are not consistent due to variation in group sizes, pre-analytical confounding factors and assay characteristics [4–7]. Furthermore, though some studies reported a relationship between the biomarkers and PD clincial progression [5, 8–10], it remains unclear how these CSF markers relate to striatal dopamine degeneration in PD [4, 11].

Vesicular monoamine transporter 2 (VMAT2) is the protein responsible for transporting dopamine, serotonin, and norepinephrine into synaptic vesicles [12]. VMAT2 imaging has been shown to be a biomarker for the monoaminergic neuron integrity in PD [13–15]. ^18^F-AV133, a positron emission tomography (PET) tracer for VMAT2 imaging that is being developed for commercial distribution, has been shown to be a promising tracer for detecting and monitoring the VMAT2 reduction in PD [13]. Therefore, the ^18^F-AV133-PET data is likely to allow us to directly assess the degeneration of monoaminergic neuron in the living PD brain. In fact, a correlation between ^18^F-AV133 uptake and cognitive impairment has been reported in Lewy body dementia patients [15].

The combination of CSF Aβ_1–42_, tau and molecular imaging has been widely studied as measures of diagnosis and disease progression in Alzheimer’s disease (AD) in recent years [16–18]. Combining molecular imaging that measures loss of synaptic function with CSF biomarkers that reflect pathologic protein accumulation may provide further insights into the mechanisms underlying neurodegenerative diseases [19]. Thus, in this study, we quantified the CSF levels of proteins for PD and AD pathology in PD patients and correlated these data with cerebral VMAT2 measured by ^18^F-AV133-PET. The ultimate goal is to improve the understanding of the interactions between pathologic protein accumulation and the loss of synaptic dopamine function in understanding motor and cognitive aspects of PD.

## Materials and Methods

### Data collection from PPMI

The anonymized and de-identified data from the Parkinson’s Progression Markers initiative (PPMI) database (http://www.ppmi-info.org/data) were downloaded in December 2014. PPMI is sponsored by The Michael J. Fox Foundation and funded by the Foundation in partnership with 16 biotech and pharmaceutical companies. It is a landmark study launched in 2010 to find biomarkers—disease indicators that are critical missing links in the search for better Parkinson’s disease (PD) treatments. The PPMI data were collected from over 33 clinical sites in 11 countries and the PPMI study was approved by the local Institutional Review Boards (IRBs) of all participating sites. Study subjects and if applicable, their legal representatives, gave written informed consent at the time of enrollment for imaging data, genetic sample collection and clinical questionnaires. Because PPMI is an observational study, research volunteers do not take any experimental drug or placebo, but agree to contribute data and samples for up to five years. The detailed information and complete list of PPMI sites’ IRBs could be found at http://www.ppmi-info.org/.

Four hundred and fifteen PD patients and 190 controls with CSF analysis from baseline were included in the current study. In addition, six PD patients and two controls were excluded due to missing p-tau or t-tau values, resulting in 409 PD and 188 controls for further analysis. All participants in the study completed an extensive evaluation battery, including clinical assessments, CSF biomarkers (α-syn, Aβ_1–42_, and tau), ^18^F-AV133 PET scans and T1 weighted structural MRI scans [20]. 10-min (2×5min) ^18^F-AV133 images acquired at 80.8 (± 2.8 SD) min post tracer injection were used in the study. As described below in more detail, the downloaded ^18^F-AV133 PET images were then processed and analyzed at both regions of interest and voxelwise levels. The relationships among ^18^F-AV133 brain uptake, CSF biomarkers, and PD severity and/or progression, approximated by the UPDRS (Unified Parkinson Disease Rating Scale) motor scores and MoCA (Montreal Cognitive Assessment) scores were examined. The study was approved by the medical ethical committees at the respective centers, and written informed consent was obtained from all participants.

### CSF Samples and Hemoglobin Tests

All CSF samples were obtained by lumbar puncture as described previously [5]. More detailed discussion of lumbar puncture procedure, CSF processing, patient acceptability and other related issues can be found in the database [20] (see also http://www.ppmi-info.org/). Because blood contamination might influence CSF α-syn concentrations, hemoglobin (Hgb) was measured in each CSF sample to monitor CSF contamination by red blood cell [1, 5]. For individuals with more than one CSF hemoglobin concentrations, the closest in date to the α-syn analysis was included [21]. Based on data from previous studies, we selected 200 ng/ml Hgb in CSF as the cut-off to exclude cases in which interpretation of α-syn might be confounded by blood contamination of CSF samples [4, 22].

### ^18^F-AV133 PET and MRI image processing

All PET and MRI images were processed using Statistical Parametric Mapping software (SPM8, Wellcome Department of Imaging Neuroscience, London, United Kingdom) and MATLAB (The MathWorks Inc.). The MRI images were normalized to standard Montreal Neurologic Institute (MNI) space using SPM8 with a high resolution MRI template provided by VBM8 toolbox [23], and the transformation parameters determined by MRI spatial normalization were then applied to the co-registered PET images for PET spatial normalization. To study the spatial and temporal changes of ^18^F-AV133 VMAT2 binding in PD progression, the PET and MRI images of the PD patients were reoriented so that the striatum contralateral to the symptomatic side was always on the left of the brain [24, 25]. The 34 regions of interest (ROIs) including cortex, striatum, and sub-striatum regions were manually drawn on the MRI template using PMOD software (PMOD Technologies Ltd., Zürich, Switzerland) in standard MNI space. The ROI of occipital cortex was used as reference tissue to calculate standardized uptake value ratio (SUVR) of ^18^F-AV133 [26, 27]. The detailed methods could be found at http://www.ppmi-info.org/: AV-133 PET Image Processing Methods for Calculation of Striatal Binding Ratio (SBR). SUVR images were calculated as PET (images)/PET (occipital) in the standard space (image volume: 121×145×121, voxel size: 1.5×1.5×15 mm in x, y, z). ROI SUVRs were then obtained by applying ROIs to SUVR images. A 3D spatial Gaussian filter of 8 mm full width at half maximum in x, y, z direction was applied to SUVR images for voxel-wise statistical analysis using SPM8.

### Statistical analysis

Analyses were performed with Statistical Package for the Social Sciences (SPSS) statistics (version 21; SPSS, Inc., Chicago, IL). Severity of motor signs in patients with Parkinson’s disease was evaluated by using the Unified Parkinson’s Disease Rating Scale motor subscale III (UPDRS-III) [8, 28]. The cognitive status was measured by Montreal Cognitive Assessment (MoCA) test [29, 30]. Comparison of demographics, VMAT2 data and CSF biomarkers between PD and healthy controls were tested with independent t tests. The relationships between the CSF biomarker and ROI SUVRs were explored by Pearson’s correlations. Complementary to ROI-based analysis, voxel-wise statistical analysis was performed using SPM8 in the study. Statistical parametric maps (SPM) were obtained for correlations between each CSF biomarker and VMAT2 by calculating the linear regression between VMAT2 SUVR images and CSF biomarker concentrations, A more stringent probability level was used to control for multiple comparisons for the results of the linear regressions (at a p-value < 0.001 for clusters > 50 voxels, corrected for cluster volume).

## Results

### CSF biomarkers at baseline

Four hundred and nine PD patients and 188 healthy controls with baseline CSF biomarkers levels from PPMI were included in this study. The means and standard deviations of demographics and clinical assessments for the PD and control groups are listed in Table 1. There were no demographic differences between groups of PD and healthy control. Consistent with previous studies [4–8, 11], significantly lower levels of α-syn were seen in PD samples in comparison to controls (p<0.001), while Aβ_1–42_ was only slightly decreased in PDs (p = 0.475). In contrast to the findings in AD [1, 5], levels of t-tau were significantly lower in PD groups than those in controls (p<0.001). Alterations in p-tau, mirrored those of t-tau, also found to be significantly decreased in the PD group when compared with controls (p = 0.014, Table 1).

**Table 1:** Demographic characteristics and clinical outcomes of HCs and Parkinson’s disease patients with CSF indicators at baseline.

Variable	PDs(n = 409)	HCs(n = 188)	p
Demographics			
Age at onset (yrs.)	61.61±9.60	61.00±11.42	0.540
M/F (n)	143:272	69:121	0.498
Clinical measurements			
UPDRS-III score	22.58±9.10	/	/
MoCA score	27.16±2.28	28.14±1.28	<0.001
UPSIT	22.42±8.18	33.82±5.35	<0.001
CSF markers (pg/mL)			
α-syn	1845.72±785.83	2201.26±1084.14	<0.001
α-syn*	1808.56±717.39	2171.62±1043.65	<0.001
Aβ_1–42_	370.47±100.37	377.62±112.98	0.475
t-tau	44.67±18.27	52.48±27.03	<0.001
p-tau	15.65±10.04	18.22±11.65	0.014

Note: Data are presented as mean±SD. HC: elderly health control; PD: Parkinson disease; UPSIT: University of Pennsylvania Smell Identification Test; UPDRS-III, unified Parkinson disease rating scale, part III; MoCA, Montreal cognitive assessment; Aβ_1–42_: β-amyloid 1–42; α-syn: α-synuclein; p-tau: phosphorylated tau; t-tau: total tau. CSF markers were analyzed using a Luminex assay.

*: α-syn excluding subjects with Hgb>200ng/mL.

Previous studies demonstrated that contamination of blood in CSF could have an influence on the level of some proteins [1, 3, 5], so CSF hemoglobin (Hgb) levels were evaluated in all CSF samples to control for this variable. Among the 409 PD subjects with CSF α-syn measurements, 19.1% were shown to have high CSF Hgb levels (>200 ng/ml). A trend toward increasing values of α-syn was observed at high CSF Hgb concentrations (r = 0.428, p<0.001) (S1 Fig). Therefore, 33 controls and 78 PD subjects with Hgb levels above the 200 ng/ml cutoff value were excluded, resulting in a total of 331 PD and 155 control subjects who were available for further α-syn analysis.

**Fig 1:**
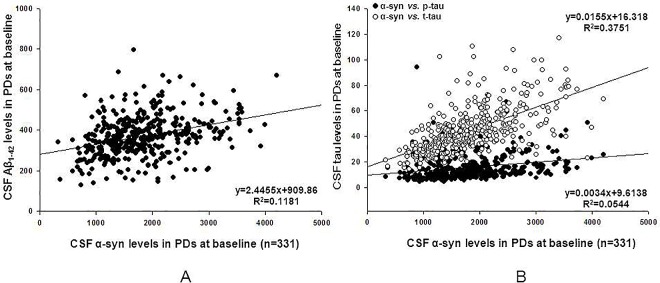
Correlations of CSF α-syn, Aβ_1–42_, t-tau, and p-tau levels in patients of Parkinson’s disease. Correlations among cerebrospinal fluid (CSF) α-syn (α-synuclein), amyloid beta 1–42 (Aβ_1–42_), total tau (t-tau) and phosphorylated tau 181P (p-tau) in Parkinson disease (PD) participants evaluated at baseline (n = 409). A significant positive correlation was found between CSF levels of α-syn and Aβ _1–42_ (A), and tau (B). Because interpretation of α-syn might be confounded by blood contamination of CSF samples, 78 subjects with Hgb levels above the 200 ng/ml were excluded from α-syn analysis. Lines indicate trends within each group as determined by linear regression.

We analyzed the correlations among the CSF biomarkers within the PDs at baseline (BL). There was a significant correlation between CSF α-syn and Aβ_1–42_ in PD participants (r = 0.326, p<0.001). CSF α-syn and p-tau, t-tau levels also displayed strong positive correlations (α-syn *vs*. p-tau: r = 0.255, p<0.001; α-syn *vs*. t-tau: r = 0.648, p<0.001) (Fig 1). However, the correlations between CSF Aβ_1–42_ and tau did not showed significance in baseline analysis (p>0.1). These results were not altered appreciably with or without controlling for baseline UPDRS motor scale and MoCA scores. As previously reported [5], no significant correlations among these CSF indicators were found in healthy controls (S2 Fig).

As aging plays a role in PD pathogenesis [28], we divided the PD subjects into two groups as early-onset PD (age at onset<50 yr) and late-onset PD (≥50 yr) to investigate the effect of aging on CSF biomarkers [31–34]. Ten PD patients and 13 controls were excluded due to missing age information, resulting 405 PD and 175 controls for analysis. No significant difference of the CSF α-syn levels, accompanied by t-tau and Aβ_1–42_ levels, were found between the early- and late-onset counterparts in the BL analysis (p = 0.206 and 0.175, 0.140, respectively, Table 2). However, when comparing the CSF biomarker correlations between early- and late-onset PD groups, we discovered that the correlations between CSF indicators were much stronger in the early-onset PD group (S1 Table). Especially, a significant correlation was observed between CSF Aβ_1–42_ and tau levels in early-onset patients (S1 Table), but as we mentioned above, no relation was found in the analysis of the PD patients as a whole.

**Table 2:** Comparison of the clinical measurements and CSF biomarker levels between early-onset and late-onset PDs.

	Age ≥ 50 yrs		Age < 50 yrs		p	
	PDs(n = 356)	HCs(n = 148)	PDs(n = 49)	HCs(n = 27)	PDs	HCs
Demographics						
Age at onset (yrs.)	64.03±7.45	64.65±7.85	44.86±4.856	50.98±5.85	<0.001	<0.001
UPDRS-III score	22.85±9.09	/	20.71±8.91	/	0.124	
MoCA	27.04 ±2.23	28.00±1.26	28.00±2.43	28.89±1.07	0.009	<0.001
CSF marker (pg/mL)						
α-syn	1868.95±788.27	2177.70±998.43	1692.88±756.52	1961.21±770.94	0.130	0.215
α-syn*	1827.91±711.81	2170.54±1088.9	1668.91±741.63	2155.55±881.03	0.206	0.341
Aβ_1–42_	366.77±99.46	381.63±113.80	388.50±95.81	343.04±73.46	0.140	0.029
t-tau	45.27±18.41	51.09±23.96	41.73±16.93	53.31±21.62	0.175	0.694
p-tau	15.19±9.65	18.68±12.18	15.72±7.99	14.84±6.28	0.672	0.018

Note: Ten PD patients and 13 controls were excluded due to missing age information, resulting 405 PD and 175 controls for analysis. Data are presented as mean±SD. HC: elderly health control; PD: Parkinson disease; UPDRS-III, unified Parkinson disease rating scale, part III; MoCA, Montreal cognitive assessment; Aβ_1–42_: β-amyloid 1–42; α-syn: α-synuclein; p-tau: phosphorylated tau; t-tau: total tau. The clinical measurements and CSF biomarker levels presented in this table were collected at baseline. Because interpretation of α-syn might be confounded by blood contamination of CSF samples, subjects with Hgb levels above the 200 ng/ml were excluded from α-syn analysis. *p* value indicates the difference between cases belong to different age groups.

*: α-syn excluding subjects with Hgb>200ng/mL.

### Correlations of CSF biomarkers with ^18^F-AV133 PET data

Among the PD patients with CSF biomarkers measurements, 22 of them had ^18^F-AV133 scans (18 males and 4 females). The mean age of this groups of patients was 64.51 yr (range: 33.7–77.3 yr) and the mean disease duration was 18.19 (range: 2–23) months. The median MoCA score and motor scale of these patients were 25.50 (range: 17–30) and 22 (range: 10–46). One limitation of the AV133-PET data in PPMI was that not all the AV133-PET was applied at the same visit, i.e. the PET images might be collected at different visits. As the objective is to analyze the correlations between CSF biomarkers and VMAT2, so the CSF data collected at the same visits as AV133-PET were used. Finally, 11 sets of data collected at baseline, 10 sets of data collected at visit 04 (visits 12 months after BL), and 1 set of data collected at visit 06 (visits 24 months after BL) were used for further analysis.

**Fig 2:**
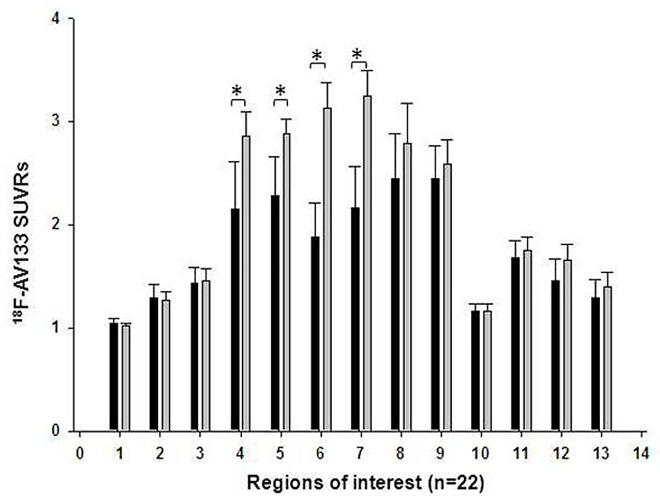
The mean ± standard deviation of ROI SUVRs of ^18^F-AV133 in healthy controls (HC) (n = 4) and Parkinson’s disease (PD) patients (n = 22). Regions of interest are numbered as: 1: orbito-frontal cortex; 2: medial-temporal cortex; 3: amygdala; 4: left caudate; 5: right caudate; 6: left putamen; 7: right putamen; 8: left ventral striatum; 9: right ventral striatum; 10: thalamus; 11: dorsal raphe nucleus; 12: substantia nigra; 13: midbrain. The SUVR in striatum subregions in PD group (n = 22) were significant lower than ones in healthy controls (n = 4) *: p<0.05 when comparing between PD and HC.

The means and standard deviations of ROI SUVRs of ^18^F-AV133 binding in PD patients were illustrated in Fig 2. There were remarkable reduced SUVRs in PD group (n = 22) in striatum sub-regions as compared to healthy controls (n = 4). The SUVRs of the amygdala, cerebellum, substantia nigra, midbrain and medial temporal lobe reduced by 5–12% from healthy controls (to 67.7–92.5% SUVR of putamen, Fig 2).

**Fig 3:**
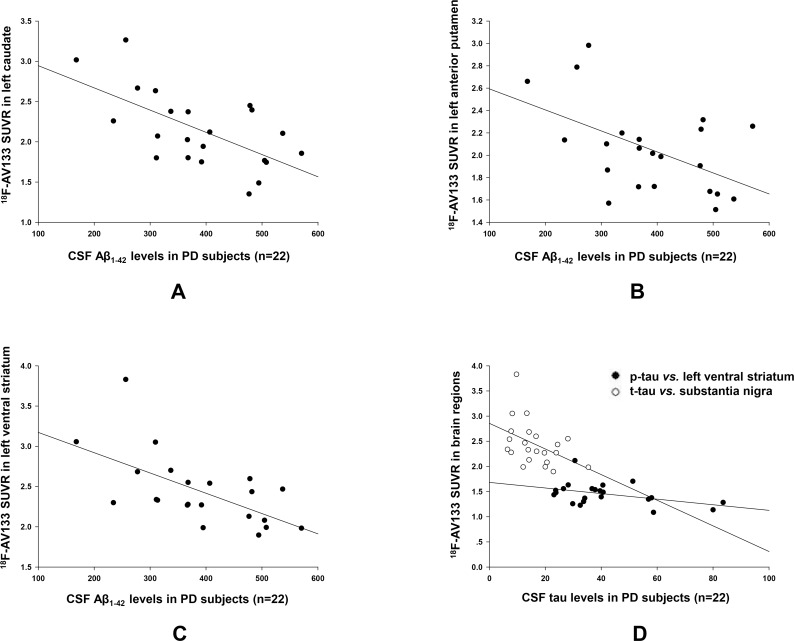
Linear correlation between CSF biomarkers and ^18^F-AV133 VMAT2 densities in Parkinson disease patients (n = 22). Typical linear plots showed that t-tau and p-tau levels were significantly correlated with ^18^F-AV133 SUVRs in the left caudate, left anterior putamen and left ventral striatum (A-C). ROI based analysis also showed linear correlations between CSF Aβ_1–42_ levels and SUVRs in substantia nigra and left ventral striatum (D).

**Fig 4:**
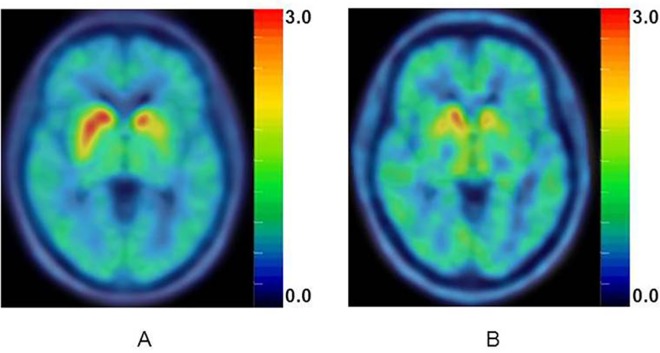
Representative ^18^F-AV133 SUVR images of PD patients with different CSF tau levels. A: a 60.7-y-old female with t-tau/p-tau of 23.8/6.5 pg/mL. B: a 58.9-y-old male with t-tau/p-tau of 29.8/13.9 pg/mL. The motor scale/MoCA score for the patient A and B were 10/30 and 35/25, respectively. ^18^F-AV133 ROI SUVRs of patient A and B were: caudate, 1.86 and 1.79; left putamen, 1.74 and 1.30; right putamen, 2.36 and 1.61; ventral striatum, 2.39 and 2.11; raphe nuclei, 1.50 and 1.73; and substantia nigra, 1.48 and 1.42, respectively.

As striatal VMAT2 binding is interpreted as reflecting integrity of the nigro-striatal dopamine system in PD dopamine is deemed to manifest the neuron integrity in PD development, we suppose that CSF biomarker levels, which reflecting pathologic protein accumulation in the neurons, might be correlated with VMAT2 evaluated with ^18^F-AV133. Results of ROI analysis showed significant negative correlations between Aβ_1–42_ and ^18^F-AV133 SUVRs. Correlations were -0.478 for posterior cingulate, -0.628 for left caudate, -0.513 for left anterior putamen, and -0.612 for left ventral striatum (p<0.001, Fig 3A–3C). Similarly, t-tau and p-tau levels were significantly negatively correlated with substantia nigra and left ventral striatum VMAT2, respectively (r = -0.429 and -0.435, p = 0.046 and 0.043, Fig 3D). No significant relationships were found between the CSF α-syn values and VMAT2. A representative SUVR image from a typical PD patient with high CSF tau levels showed higher VMAT2 SUVRs and better UPDRS motor scale/MoCA score as compared to patient of low CSF tau concentrations (Fig 4).

**Fig 5:**
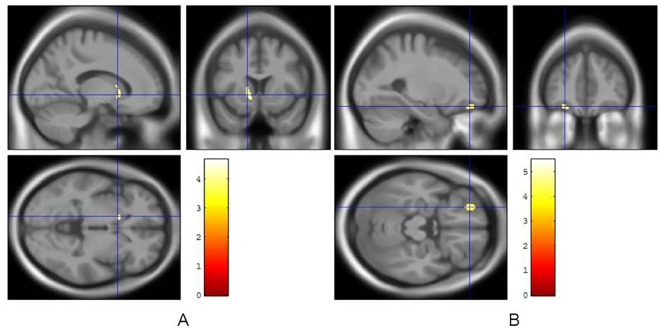
SPM map. Significant negative correlations between cerebral VMAT2 density and CSF Aβ_1–42_ (A) and p-tau (B) levels normalized scores with adjustment for age and sex of the PD patients. Significant clusters are displayed with slices of MRI templates in axial, coronal and sagittal orientations. p-value < 0.001 at the voxel level for clusters>50 contiguous voxels (corrected for cluster volume).

Results from voxelwise statistical analysis showed that a single large cluster of 107 voxels (peak T = 4.46 at -12 mm, 14 mm, -3 mm in x, y, z) mainly involving the left caudate was negatively correlated with CSF Aβ_1–42_ level (Fig 5A). SPM map of CSF Aβ_1–42_ levels correlations also revealed three small clusters (105, 51, and 99 voxels) that included the parahippocampal gyrus, insula and temporal lobe (Fig 5A). In addition, SPM8 analysis detected negative correlations between CSF p-tau level and clusters of 118/66 voxels (peak T = 5.48 at -26 mm, 44 mm, -18 mm in x, y, z; T = 4.30 at 57 mm, -12 mm, 13 mm in x, y, z) mainly including superior frontal gyrus and transverse temporal gyrus (Fig 5B).

### Comparison of CSF biomarkers in PDs with possible cognitive decline

**Fig 6:**
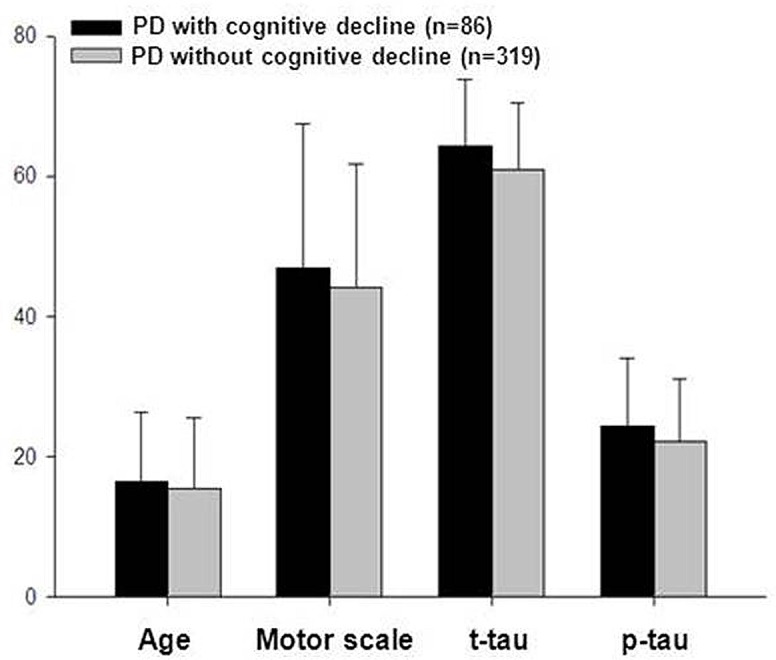
Comparisons of the clinical outcomes and CSF tau levels in PDs with possible cognitive deficit. There was a significant difference of the mean age among the patients of Parkinson’s disease with, or without cognitive decline (PD-D, or PD-ND) (60.90±9.52 *vs*. 64.24±9.44 yr, p = 0.004), which further proved the vital role aging plays in the cognitive decline in PD development. As we expected, the motor severity increased with the cognitive decline in the patients (22.10±8.92 *vs*. 24.37±9.51, p = 0.05). The t-tau and p-tau levels increased simultaneously with the cognitive deficit progression in PD. The p-tau and t-tau levels increased from 15.43±10.11 and 44.05±17.62 pg/ml in PD-ND, to 16.47±9.74 and 46.96±20.30 pg/ml in PD-D, though the difference did not reach significance (p>0.1).

As CSF biomarkers were mostly correlated with the ^18^F-AV133 SUVRs in brain regions known for cognitive function, we speculated that these proteins might be related to the cognitive function in PD patients. Subsequently we analyzed the relationship of these proteins with cognitive deficits that indexed by MoCA scores in PD [29, 30]. Ten PD patients were excluded due to missing MoCA information, resulting 405 PD for analysis. These patients were divided into cognitively normal (PD-ND, MoCA>25, n = 319) and cognitive impaired PDs (PD-D, MoCA≤25, n = 86). Thus, a tendency for an increased CSF Aβ_1–42_ level in PD-D when comparing with PD-ND, although not statistically significant, was noted (388.89±115.18 vs. 365.52±95.40 pg/ml, p = 0.087). Analysis of tau levels also showed slightly increased tau levels in cognitive deficit PDs (Fig 6). No obvious difference was shown when comparing the CSF α-syn levels in PD-D (1823.87±819.30 pg/ml) and PD-ND (1851.59±776.49 pg/ml, p = 0.779). Finally, no apparent linear correlation was found between CSF biomarkers and disease progression of PD patients in the sub-groups (data not shown).

## Discussion

Our main findings were that: (i) as expected, CSF α-syn were significantly lower in PD as compared with controls. Aβ_1–42_ was also decreased in PDs, but the effect was not significant. In contrast with elevated CSF t-tau and p-tau in Alzheimer disease, CSF tau were significantly lower in PD; (ii) CSF α-syn positively correlated with Aβ_1–42_ and tau levels in PD participants, and the correlation was more greater in the early-onset PDs; (iii) CSF Aβ_1–42_ and tau levels were inversely correlated with VMAT2 SUVRs in cortical brain areas associated with cognitive dysfunction.

Several studies, including the present one, have shown that CSF α-syn is significantly decreased in patients with PD compared with controls [4–6, 8, 11]. The reduced α-syn levels in CSF, most likely reflect the balance of the accumulation α-syn fibrillary aggregates in cortex and α-syn tangles secondary to the neurodegeneration in PD [35]. This has been suggested as a helpful biomarker for PD [4, 8, 36]. However, most studies indicated that CSF α-syn alone did not provide relevant information for PD diagnosis [37], and cross-sectional studies failed to find correlations between CSF α-syn levels and PD severity progression [11, 35], as we found no linear correlations between α-syn and progression of motor/cognitive symptoms in our study. We speculate that this phenomenon might be caused by the complication of the molecular pathogenesis of PD [38]. A growing number of studies support the hypothesis that the severity of PD is associated with the interactions between tauopathies and synucleinopathies [39, 40], so the altered α-syn levels alone may not stand for the synergistic effects of the “triple brain amyloidosis” that leading to the neurodegeneration in PD [39, 40]. In support of this, recent study found that a combination of measures of cortical α-syn, tau, and Aβ pathologies in the regression model was more predictive of cognitive decline in PD than any single marker alone [40].

In addition to significantly lowered α-syn level in PD patients, we also found levels of tau decreased in PD than in controls. It is suggested that CSF Aβ, tau, and α-syn may interact synergistically to promote the accumulation of each other in the cortex, and thus contribute to the pathological cascade of PD [40]. So the interpretation of the PD specific CSF tau decrease is also attributed to the α-syn accelerated depositing of tau. Indeed, there is evidence that certain α-syn fibrils might induce aggregation of tau by cross-seeding [41, 42]. Furthermore, α-syn has been shown to contribute to the phosphorylation of tau in various mouse and human models [42]. Our analysis of the correlations among CSF markers confirmed the strong correlation between Aβ, tau, and α-syn levels (Fig 1).

The direct interactions among CSF biomarkers in Parkinson’s disease have been repeatedly reported [39, 40]. In the current study, we investigated the impact of age at onset of the disease on the CSF marker interactions, as it is well known that patients with onset of Parkinson's disease fewer than 50 years of age may have a more favorable prognosis than those whose symptoms begin in a later age [34, 43]. Our results discovered that the correlations among the CSF markers were much stronger in the early-onset PDs (age at onset<50 yr), when compared with the late-onset subjects (≥50 yr, S1 Table). When excluding the patients ≥50 yr, a significant correlation between CSF Aβ_1–42_ and tau levels showed up, while no relation was found in the analysis of the whole group. One potential testable hypothesis could be that the aggregations and deposition secondary to cross-seeding of the pathologic proteins may be less toxic than the oligomers composed of pure tauopathies, or synucleinopathies, as the amyloid oligomers have emerged as the most toxic species of amyloid-β and tau oligomers may be more closely related to tau neurotoxicity than the presence of the tangles themselves [44, 45].

A relationship between β-amyloidopathy and cognitive functions in PD has been reported by in vivo imaging and postmortem studies [46]. The main finding of this study is that there is an inverse correlation between CSF Aβ_1–42_ levels and VMAT2 SUVRs in brain areas known for emotion and cognitive function (caudate, parahippocampal gyrus, insula and temporal lobe), indicating that the cognitive status might be inversely correlated with CSF Aβ_1–42_ in PD patients. Although not statistically significant, our observation noted a tendency for an increased CSF Aβ_1–42_ level with the worsening of the cognitive status in PD. This is counterintuitive because, as previously reported, the CSF levels of Aβ_1–42_ were lower in patients with PD compared to healthy controls in most studies [2, 3]. The decrease of CSF Aβ_1–42_ in PD may be caused by the progressive deposition of Aβ_1–42_ in the brain, or by a decreased production of Aβ_1–42_ by neurons [47, 48]. Thus, one would expect PD progression to be associated with a further decrease, rather than an increase, in Aβ_1–42_. However, our study showed an inverse relationship between neuronal dysfunction and CSF Aβ_1–42_ levels. Actually, similar findings had been reported by Bouwman et al. They discovered a significant increase in CSF Aβ_1–42_ over baseline levels in patients with probable AD [49]. They concluded that, although CSF Aβ_1–42_ is reduced compared to controls, this may represent a compensatory response and levels might increase with greater cognitive impairment to suggest AD diagnosis [49, 50]. One interpretation of this increase could be that Aβ oligomers, more toxic and detectible in CSF, were released from the damaged neurons with the progression of the disease [51]. Arlt et al also reported a negative correlation between cerebral glucose metabolisms in the precuneus/posterior cingulate with CSF Aβ_1–42_ concentrations [50]. This CSF Aβ_1–42_ increase accompanied by a decrease of neuron glucose metabolism might be better explained by the progressive loss of living neurons which causing constitutively producing and secreting β-amyloid protein into CSF.

The levels of CSF tau showed an inverse correlation with VMAT2 SUVRs in substantia nigra and left ventral striatum (r = -0.429 and -0.435, p = 0.046 and 0.043). This is quite similar to the results from the analysis of glucose metabolism and CSF markers [52, 53]. Ceravolo et al. targeted the relationship between CSF t-tau and p-tau and glucose metabolism in a cohort of 28 subjects with probable AD and showed a significant negative correlation between both t-tau and p-tau and glucose metabolism bilaterally in the temporal lobe, the parietal lobe, and the entorhinal/hippocampal region [52]. As expected, our subsequent analysis of the CSF tau levels and cognitive status in PD patients showed that tau concentrations increased with cognitive decline in PDs (Fig 6). This increase in CSF tau levels parallel PD progression may be secondary to neuronal damage and cell death (i.e., tau being released from damaged cells).

## Conclusions

This pilot study provides imaging evidence that CSF Aβ_1–42_ and tau levels negatively correlated with VMAT2 SUVRs in brain regions associated with cognitive dysfunction in PD, indicating a relationship between these pathologic proteins and dopamine degeneration in PD. Considering the high heterogeneity in PD development, we realized one main limitation of the study is that the CSF biomarker and VMAT2 correlation study was based on only 22 patients. If supported in larger studies the VMAT2 PET measures could relate to subsequent cognitive dysfunction especially in those early onset PD subjects under 50 years. The role of this relationship in PD progression, especially in patients with cognitive decline, can be studied longitudinally with the ongoing PPMI project to identify the earliest neurobiological changes associated with cognitive decline in PD.

## Supporting Information

S1 Fig Correlations between CSF α-syn and hemoglobin at high CSF Hgb concentrations. CSF α-syn and CSF Hgb concentrations were significantly correlated in Parkinson’s disease subjects with Hgb levels above the 200 ng/ml (r = 0.428, p = 0.000, n = 78).
(TIF)

S2 Fig Correlations among CSF indicators in healthy controls. Correlations between cerebrospinal fluid (CSF) α-syn (α-synuclein) and amyloid beta 1–42 (Aβ_1–42_) (A), α-syn and total tau (t-tau), phosphorylated tau 181P (p-tau) (B) in healthy controls evaluated at baseline (n = 188). No significant correlations were found among these indicators. Because interpretation of α-syn might be confounded by blood contamination of CSF samples, 33 subjects with Hgb levels above the 200 ng/ml were excluded from α-syn analysis. Lines indicate trends within each group as determined by linear regression.
(TIF)

S1 Table The correlations among CSF biomarkers in early- and late-onset PD groups. (DOCX)
