# Supplementary figures and images for: CSF Biomarkers and Its Associations with 18F-AV133 Cerebral VMAT2 Binding in Parkinson’s Disease—A Preliminary Report
Source: PLoS One. 2016 Oct 20;11(10):e0164762. doi: 10.1371/journal.pone.0164762 (PMC5072678; doi:10.1371/journal.pone.0164762)

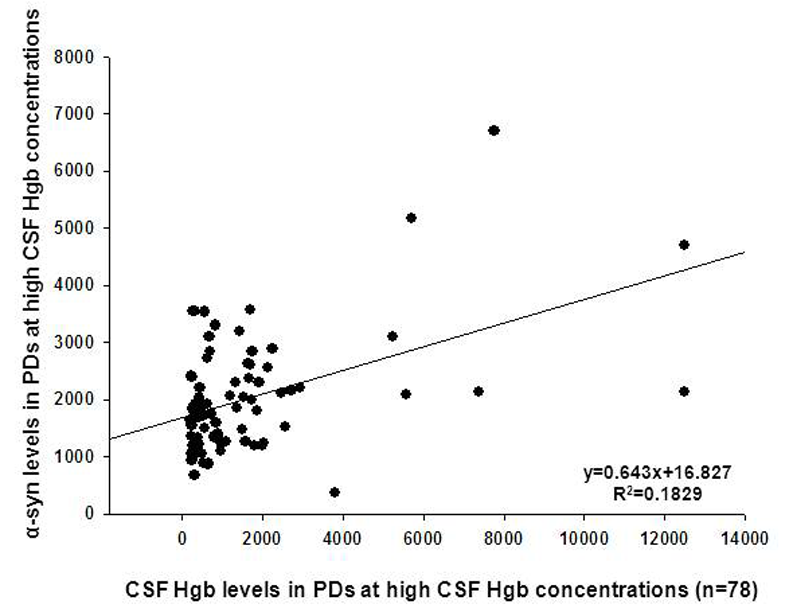

Supplement: S1 Fig — CSF α-syn and CSF Hgb concentrations were significantly correlated in Parkinson’s disease subjects with Hgb levels above the 200 ng/ml (r = 0.428, p = 0.000, n = 78). (TIF) [file pone.0164762.s001.tif]

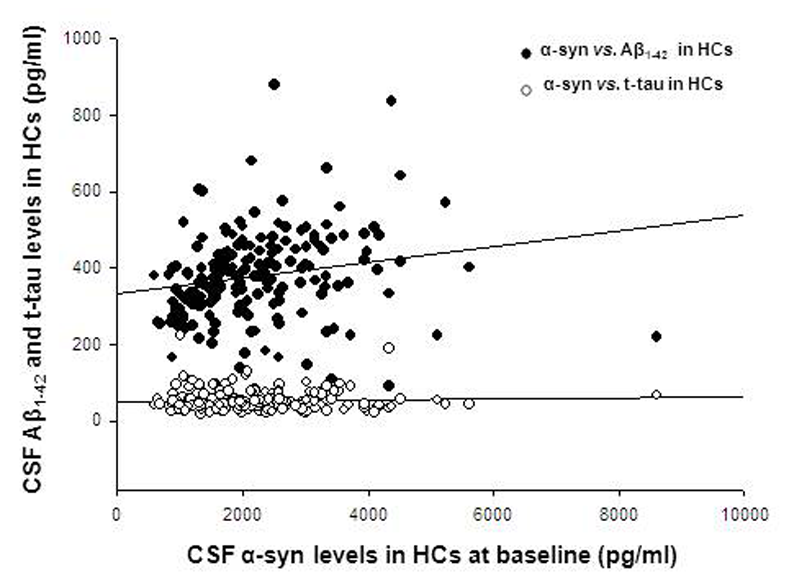

Supplement: S2 Fig — Correlations between cerebrospinal fluid (CSF) α-syn (α-synuclein) and amyloid beta 1–42 (Aβ1–42) (A), α-syn and total tau (t-tau), phosphorylated tau 181P (p-tau) (B) in healthy controls evaluated at baseline (n = 188). No significant correlations were found among these indicators. Because interpretation of α-syn might be confounded by blood contamination of CSF samples, 33 subjects with Hgb levels above the 200 ng/ml were excluded from α-syn analysis. Lines indicate trends within each group as determined by linear regression. (TIF) [file pone.0164762.s002.tif]
