# Supplementary material for: CSF Biomarkers and Its Associations with 18F-AV133 Cerebral VMAT2 Binding in Parkinson’s Disease—A Preliminary Report
Source: PLoS One. 2016 Oct 20;11(10):e0164762. doi: 10.1371/journal.pone.0164762 (PMC5072678; doi:10.1371/journal.pone.0164762)
Supplement: S1 Table — (DOCX) [file pone.0164762.s003.docx]

**S1Table The correlations among CSF biomarkers in early- and late-onset PD groups.**

| CSF marker (pg/mL) | Aβ_1-42_ | | |
| --- | --- | --- | --- |
|  |  | Age ≥ 50 yrs | Age < 50 yrs |
| α-syn | Pearson r | 0.324 | 0.522 |
|  | *p* | 0.000 | 0.000 |
| t-tau | Pearson r | 0.047 | 0.426 |
|  | *p* | 0.381 | 0.002 |
| p-tau | Pearson r | 0.008 | 0.313 |
|  | *p* | 0.887 | 0.025 |

Note: Correlations among cerebrospinal fluid (CSF) amyloid beta 1-42 (Aβ_1-42_) and α-syn (α-synuclein), total tau (t-tau), phosphorylated tau 181P (p-tau) were measured in Parkinson’s disease (PD) subjects belong to early-onset (n=49) and late-onset (n=356) sub-groups in this table. As CSF α-syn concentrations might be confounded by blood contamination, 78 subjects with Hgb levels above the 200 ng/ml were excluded from α-syn analysis. The relationship of CSF Aβ_1-42_ vs. α-syn, p-tau, or t-tau was closer in the early-onset PDs, when comparing with the subjects in late-onset sub-group in the baseline analysis.
